# Comparison of bioimpedance equations and dual-energy X-ray for assessment of fat free mass in a Chinese dialysis population

**DOI:** 10.1080/0886022X.2023.2182131

**Published:** 2023-03-01

**Authors:** Yanna Dou, Afang Li, Gangrong Liu, Peipei Wang, Bei Zhang

**Affiliations:** a Department of Nephrology, The First Affiliated Hospital of Zhengzhou University, Zhengzhou, Henan, China; b Department of Bone Mineral Density, The First Affiliated Hospital of Zhengzhou University, Zhengzhou, Henan, China

**Keywords:** Bioimpedance, chronic kidney disease (CKD), dialysis, nutrition

## Abstract

**Purpose:**

Bioelectrical impedance analysis (BIA) is simple, noninvasive, inexpensive and frequently used for estimating fat free mass (FFM). The aims of this study were to evaluate the applicability of different BIA equations on FFM in Chinese subjects, and to compare the difference in hemodialysis and peritoneal dialysis patients with healthy controls respectively.

**Methods:**

Dialysis patients and healthy adults were enrolled in this study, and the subjects were matched by age, gender, and the minimum sample size in each group was calculated using PASS. FFM estimated by BIA was calculated using equations of Kyle, Sun SS and Segal, and TBW/0.73. Dual-energy X-ray absorptiometry (DXA) method was set as reference method. Pearson’s correlation and Bland-Altman analysis were used to test the validity of the BIA equations.

**Results:**

50 hemodialysis (HD) patients, 52 peritoneal dialysis (PD) patients and 30 healthy adults aged 22–67 y were included in this study. Age, height, weight, BMI and gender did not differ significantly among HD, PD patients, and healthy controls (*p* > 0.05), but BIA parameters were quite different (*p*＜0.01). Bland-Altman analysis showed that in healthy volunteers, all equations showed good agreement with DXA measured. For dialysis patients, the FFM predictions of different equations showed differences between HD and PD patients, and the equations seemed more applicable for HD patients.

**Conclusion:**

The equations developed by healthy subjects might be not appropriate for dialysis patients, especially peritoneal dialysis patients. It is recommended to develop a specific BIA equation from dialysis population.

## Introduction

Malnutrition is a common phenomenon in chronic kidney disease (CKD), especially in dialysis patients, and is thought a risk factor for increased all-cause mortality and cardiovascular death [1–3]. This makes it necessary to have an appropriate method for nutritional assessment of this population. Dual energy X-ray absorptiometry (DXA) is considered a reference method for estimating the whole body composition [4], but it requires specially-trained operators and the cost is high, making it difficult to monitor the nutritional status of dialysis patients. In recent years, bioelectrical impedance analysis (BIA) has been developed for use in estimating body composition in different population and is noninvasive, relatively simple and inexpensive [5–7]. In Carlo Basile’s research, they used BIA to develop an equation for dry weight in hemodialysis patient [8], and Rong-shao Tan predicted protein-energy wasting in maintenance hemodialysis patients using phase angle from BIA [9]. However, there has not been a specific equation estimating nutrition status of dialysis patients. More importantly, the fluid distribution of hemodialysis (HD) and peritoneal dialysis (PD) was very different [10]. The equations used to estimate fat mass (FM) and fat free mass (FFM) are usually developed from healthy subjects [11], the performance of these BIA equations might be appropriate for the specific population and influenced by different diseases, ethnicity [12,13]. Hence, in this study, we evaluated the applicability of different BIA equations in HD, PD dialysis patients and healthy adults compared to standard DXA scanning, respectively.

## Materials and methods

### Subjects

Hemodialysis (HD) or peritoneal dialysis (PD) patients were recruited from those who received hospitalization or outpatient treatment in the First Affiliated Hospital of Zhengzhou University from September 2018 to August 2019. Patients who had been undergoing dialysis treatment for at least 3 months and were 18–80 years were included, but those with pacemakers or amputation were excluded. Healthy ambulatory subjects who were 18-80 years were recruited from those who undergone a physical examination at the physical examination department of the First Affiliated Hospital of Zhengzhou University from September 2019 to October 2019. All the subjects were matched by age and gender, and the minimum sample size in each group was calculated using PASS. Approval for the study was obtained from the Ethics Committee of Zhengzhou University, ethical review number: 2018-KY-36, and written informed consent was obtained from each subject. For HD patients, measurements were performed one day after HD treatments, and for PD patients, measurements were performed with peritoneal cavity empty. All subjects reported to the study following a minimum of an eight-hour fast before the testing session.

### Anthropometric measures

All the subjects were measured the height and weight using ultrasonic height and weight meter (OMRON, Japan), with the subjects in light clothing and standing erect, arms hanging freely at their sides.

### Dual-energy X-ray absorptiometry

The reference test for assessment of body composition was DXA (Hologic Inc., USA), measurements were performed with subjects in the supine position, arms at their sides, palms down, legs rotated inward about 25 degrees, and then maintained this position during the X-rays scanned through the whole body. Lean mass, bone mineral mass and fat mass were derived using software provided by the manufacturer since they had different attenuation characteristics, and FFM was calculated as the sum of lean mass and bone mineral mass.

### Bioelectrical impedance analysis

The bioelectrical impedance analyzers can be divided into single-frequency (SF-BIA) and multi-frequency (MF-BIA) basing on the used current frequency. MF-BIA devices can use frequencies ranging from 5 to 1000 kHz, and typically use Cole molding [14] to predict intracellular and extracellular water independently. While SF-BIA devices use a single 50 kHz current to calculate the resistance (R) and reactance (Xc), and these values are applied to specific regression equations for predicting body composition.

In this study, BIA measurements were performed using a multi-frequency bioelectrical impedance analyzer (Xitron 4200, USA). Practically, the skin was cleaned with 75% alcohol, four electrodes were placed on the non-access side of the patients and dominant side of healthy subjects, and each two electrodes were placed on the dorsal surfaces of the hand and foot proximal to the metacarpal-phalangeal and metatarsal-phalangeal joints separately. Measurements were taken after the subject lay supine for a minimum of 5 min, with arms and legs abducted from the body. R and Xc values were extracted at 50 kHz, and used to predict FFM as previously described.

### Selection of predictive equations of BIA

Typically, the fraction of FFM as total body water (TBW) is relative stable at 0.73 [15], TBW includes both intracellular and extracellular water. Therefore, for the MF-BIA devices, FFM_-TBW_ = TBW/0.73.

SF-BIA predictive equations were selected adopting these criteria:(a) the age of subjects was compatible with our study; (b) samples involved both male and female subjects; and (c) BIA equipment was consistent with our study (including a frequency of 50 kHz). We selected three equations from the previously published literatures:

$$\text{Kyle}\;\text{et}\;\text{al}.\;[16]\;\;：{\text{FFM}}_{-\text{Kyle}}=-4.104+\left(0.518*H^{2}/R_{50}\right)+\left(0.231*W\right)\;\;+(0.130*{\text{Xc}}_{50)}+\left(4.229*\text{sex}\right)$$

$$\text{Sun}\;\text{SS}\;\text{et}\;\text{al}.\;[17]\;：\text{male}:{\text{FFM}}_{-\text{SS}}\;=-10.68+\left(0.65*H^{2}/R_{50}\right)+\left(0.26*W\right)\;\;+\left(0.02*R_{50}\right);\;\text{female}:{\text{FFM}}_{-\text{SS}}\;=-9.53+(0.69*H^{2}/R_{50)}+\left(0.17*W\right)\;\;+\left(0.02*R_{50}\right)$$

$$\text{Segal et al}.\;[18]：\mathrm{male}:{\mathrm{FFM}}_{-\mathrm{Segal}}\;=22.66827+(0.00132\text{ * }H^{2})-(0.04394*R_{50})\;\;+(0.30520\text{ * W})-(0.16760\text{ * age});\mathrm{female}:{\mathrm{FFM}}_{-\mathrm{Segal}}\;=14.59453+(0.00108\text{ * }H^{2})–(0.02090\text{ * }R_{50})\;\;+(0.23199\text{ * W})-(0.06777\text{ * age}),$$

where R_50_=resistance at 50 kHz, Xc_50_= reactance at 50 kHz, H = height, W = weight, and sex is coded 0 for female and 1 for male.

### Statistical Analysis

Statistical analysis was carried out with SPSS 22.0 (IBM, Chicago, IL, USA) and GraphPad Prism 8.0 (GraphPad Software Inc., San Diego, CA, USA). The minimum sample size was estimated by PASS. Descriptive statistics were given as means ± standard deviation (SD), and categorical variables were given as numbers and percentages. One-way analysis of variance was used to compare the differences of patients and healthy adults in anthropometric measures and BIA parameters, and chi-square analysis was used for the comparison of categorical variables. Pearson’s correlation was conducted to determine the consistency between the FFM estimated by BIA equations and DXA, and Bland–Altman analysis was used to compare the agreement between the two measurements. *p*<0.05 was considered to be statistically significant.

## Results

50 hemodialysis (HD) patients, 52 peritoneal dialysis (PD) patients and 30 healthy adults aged 22-67 y were included in this study, the anthropometric, BIA characteristics were shown in Table 1. Age, height, weight and BMI did not differ significantly among HD, PD patients and healthy controls (*p* > 0.05). Moreover, there was no significant gender difference (*p* = 0.46). R_50_, Xc_50_ were significantly lower and H^2^/R_50_ was significantly higher in patients than healthy participants (*p*<0.01).

**Table 1.** Comparison of anthropometric, BIA characteristics and clinical data in dialysis patients and healthy adults.

Parameters	HD	PD	Healthy adults	*p*
	(*n = 50*)	(*n = 52*)	(*n = 30*)	
Age (y)	45.80 ± 11.37	46.35 ± 11.10	44.13 ± 11.64	0.69
Male (*n*,%)	33 (66.0)	28 (53.8)	18 (60.0)	0.46
Female (*n*,%)	17 (34.0)	24 (46.2)	12 (40.0)	
Height (cm)	166.37 ± 9.01	165.49 ± 8.43	167.73 ± 8.13	0.52
Weight (kg)	69.01 ± 14.21	66.11 ± 11.59	69.95 ± 12.14	0.34
BMI (kg/m^2^)	24.81 ± 4.00	24.03 ± 3.07	24.88 ± 4.25	0.48
R_50_ (Ω)	484.25 ± 96.07	418.22 ± 85.75	533.61 ± 75.00	0.00
Xc_50_ (Ω)	47.16 ± 13.00	36.03 ± 12.26	56.69 ± 7.11	0.00
H²/R_50_	60.20 ± 16.10	68.92 ± 18.60	53.95 ± 9.55	0.00

H: height; BMI: body mass index; R_50_: resistance at 50 kHz; Xc_50_: reactance at 50 kHz.

In patients, the FFM predicted by BIA_-Sun SS_ equation were significant higher than DXA measured both in HD and PD patients (*p*<0.05; Table 2). In healthy volunteers, BIA_-Kyle_, BIA_-Sun SS_, BIA_-Segal_ and BIA_-TBW_ showed no significant differences in estimated FFM values with DXA measured (*p* > 0.05; Table 2). However, FFM estimated by BIA equations correlated well with DXA measurement (*r* between 0.95 to 0.98) in all participants.

**Table 2.** Evaluation of FFM estimated by BIA equations against DXA as reference method.

	Mean ± SD	*p * ^a^	*r * ^b^
HD *(n = 50)*			
FFM_-DXA_	50.30 ± 10.69		
BIA			
FFM_-Kyle_	51.94 ± 11.60	0.46	0.98
FFM_-Sun SS_	55.29 ± 12.39	0.03	0.98
FFM_-Segal_	51.02 ± 10.39	0.73	0.97
FFM_-TBW_	50.72 ± 11.11	0.85	0.95
PD *(n = 52)*			
FFM_-DXA_	50.39 ± 9.08		
BIA			
FFM_-Kyle_	53.83 ± 12.58	0.11	0.97
FFM_-Sun SS_	58.77 ± 13.59	0.00	0.96
FFM_-Segal_	51.58 ± 10.21	0.53	0.96
FFM_-TBW_	53.08 ± 11.30	0.18	0.95
Healthy volunteers(n = 30)			
FFM_-DXA_	48.36 ± 8.79		
BIA			
FFM_-Kyle_	49.91 ± 8.53	1.00	0.96
FFM_-Sun SS_	52.11 ± 8.46	0.64	0.97
FFM_-Segal_	49.99 ± 8.23	1.00	0.97
FFM_-TBW_	48.37 ± 7.82	1.00	0.96

Estimates of FFM are given as kg; ^a^significance of the FFM difference among DXA and different BIA equations methods; ^b^Pearson correction between BIA equation and DXA values. *Abbreviations*: BIA: bioelectrical impedance analysis; DXA: dual energy X-ray absorptiometry; FFM: fat free mass.

**Figure 1.**
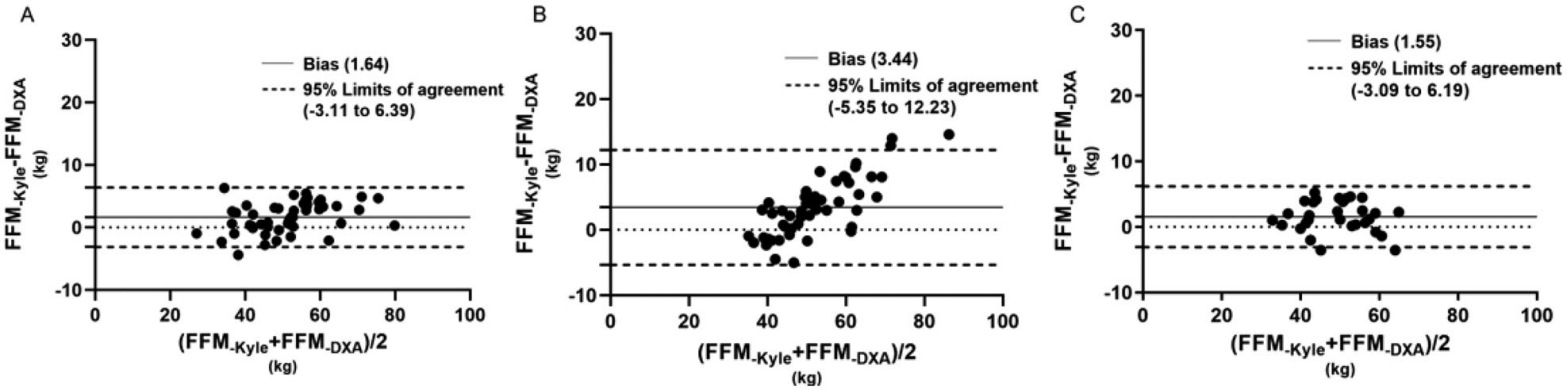
Bland–Altman plots for hemodialysis patients (A), peritoneal dialysis patients (B) and healthy adults (C) of FFM estimated by BIA-Kyle and DXA methods.

**Figure 2.**
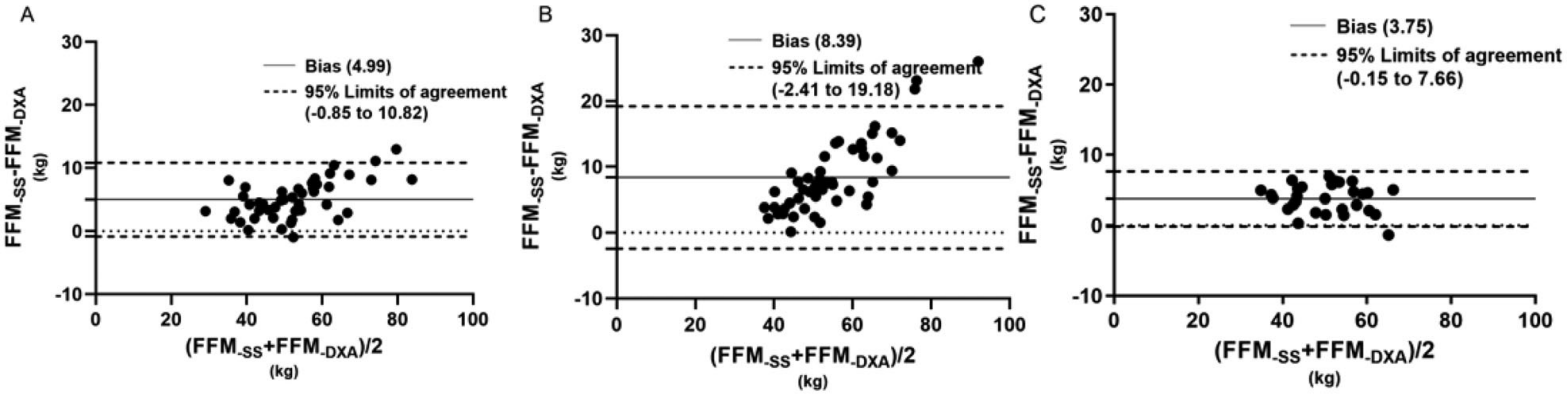
Bland–Altman plots for hemodialysis patients (A), peritoneal dialysis patients (B) and healthy adults (C) of FFM estimated by BIA-Sun ss and DXA methods.

**Figure 3.**
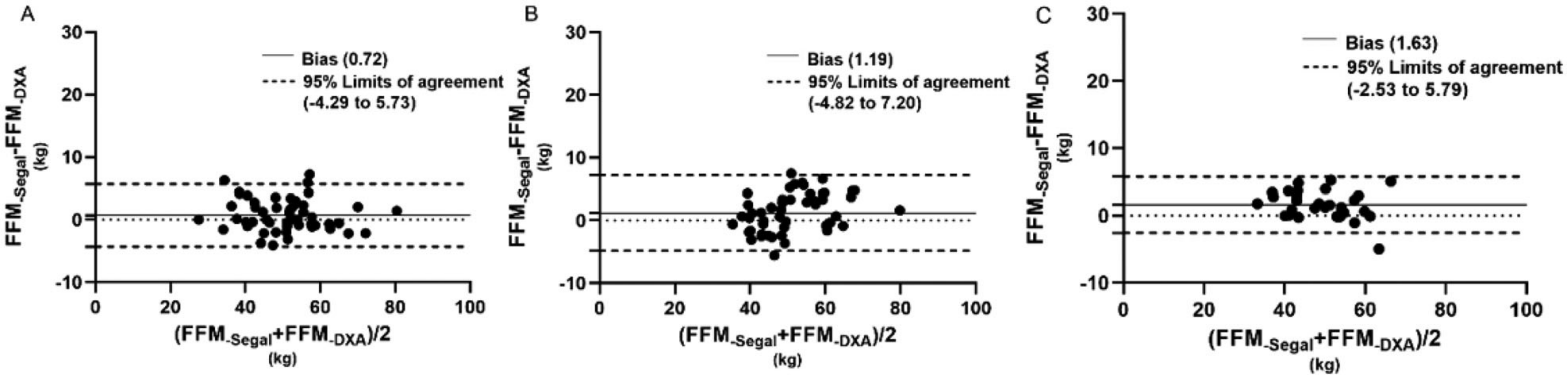
Bland–Altman plots for hemodialysis patients (A), peritoneal dialysis patients (B) and healthy adults (C) of FFM estimated by BIA-Segal and DXA methods.

**Figure 4.**
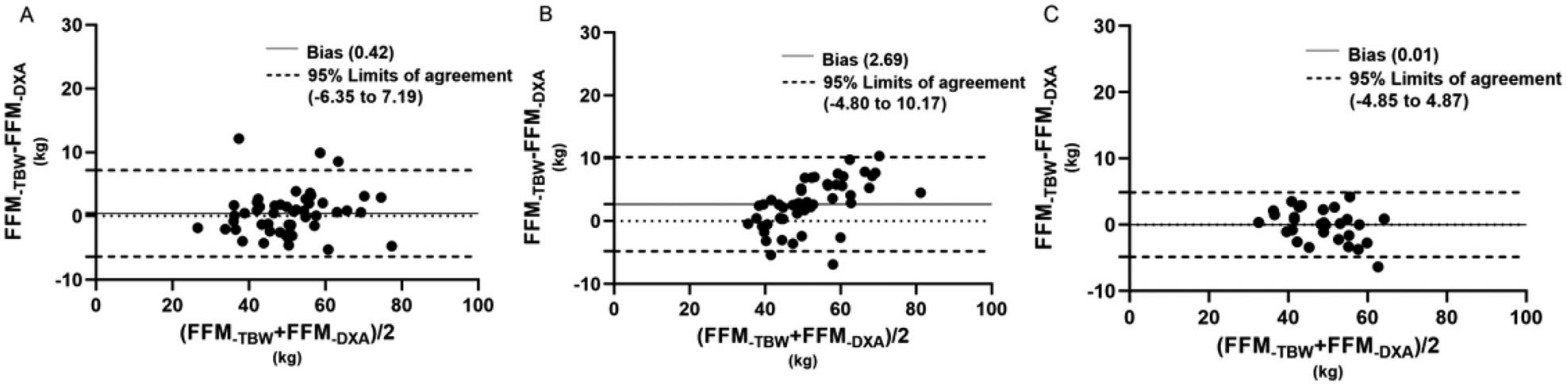
Bland–Altman plots for hemodialysis patients (A), peritoneal dialysis patients (B) and healthy adults (C) of FFM estimated by BIA-TBW and DXA methods.

Figures 1–4 illustrated the tendencies by Bland-Altman plots, and revealed the applicability of different equations for the subjects. For healthy volunteers, all equations showed good agreement with DXA measured. For dialysis patients, the FFM predictions of different equations showed differences between HD and PD patients. In Kyle equation, the HD patients showed a good agreement with DXA measured, the bias was 1.64, with 95% limits of agreement (LOA) of −3.11 to 6.39. However, the values of PD patients showed a tendency that BIA_-Kyle_ overestimated the FFM as the weight increases gradually, the bias and 95% LOA (3.44, −5.35 to 12.23) were both higher than HD patients. In Sun SS equation, the predictions for HD patients and PD patients showed a similar tendency, but the bias and 95% LOA of PD patients (8.39, −2.41 to 19.98) were higher than HD patient (4.99, −0.85 to 10.82). In Segal equation, both HD and PD patients showed good agreement with DXA measured, the bias and LOA were lower (0.72, −4.29 to 5.73, 1.19, −4.82 to 7.20 respectively) than other equations. In TBW equation, the HD patients also showed a better agreement than PD patients, the bias and LOA were 0.42, −6.35 to 7.19, 2.69, −4.80 to 10.17, respectively.

## Discussion

We examined three single frequency BIA equations and multifrequency bioelectrical impedance estimating FFM in individuals, the Segal equation showed the smallest mean difference and the best agreement with DXA, these results suggest that the Segal equation may be superior to the other equations for estimating FFM in Chinese dialysis patients. In this study, the subjects ranged in age from 22 to 67 y, while the Kyle, Sun ss and Segal equations were developed from the subjects aged 22–94 y, 12–94 y and 17–62 y respectively. The age of subjects in Segal equation was the most consistent with our study, and it provided a parameter of age, whereas, the other two equations included adults aged 80 and over. We thought that prediction equations developed from older subjects were not applied to other groups. Our results are in accordance with a published literature by Vermeiren et al. [19], including 189 older adults aged 80-95 y, and underscoring that selection of BIA equation has significant implications for the accuracy of different age groups.

Due to the absence of renal function and the intermittency of treatment, the fluid status of dialysis patients is varying across a week. Fluid volume of patients on hemodialysis (HD) falls during treatments and rises between dialysis sessions, which ranges from fluid depletion to fluid overload [20]. Although peritoneal dialysis (PD) provides a continuous ultrafiltration, fluid overload is quite common, and may cause adverse cardiovascular outcomes [21]. In our study, FFM of healthy volunteers derived from Kyle, Sun ss equations and TBW had a better agreement with DXA than dialysis patients, more exactly, the predictions of HD patients were better than PD patients. It might be caused by different body fluid status in different kinds of dialysis, in our clinical observations, PD patients were more likely to have lower limb edema or even generalized edema, as Yilmaz’s research revealed that overhydration/extracellular ratio is higher in the PD patients than post-HD patients [22]. We thought that equations developed from healthy adults might be not appropriate for dialysis patients with abnormal hydration, and it is essential to select a specific equation for dialysis patients, this is consistent with a report of body composition assessment in HD patients by Bross et al. [23].

The Kyle equation were developed in European populations, and the others were developed in American populations, while the subjects were from different sites. In this study, the subjects were all from the central part of China, and the results revealed that whether in healthy adults or in patients, the mean differences of FFM estimated from these equations with DXA were different, which was consistent with a research by Nightingale et al. reporting that ethnic-specific equations could provide better estimate of ethnic differences and generic equations could misrepresent the differences [24]. In recent years, accumulating researches suggest that body composition differs between ethnic groups [25,26]. For instance, Deurenberg et al. reviewed that Asian populations had a higher abdominal adiposity than European populations at the same BMI[27].

The study had several limitations, a larger sample would allow better estimates of different equations, and a large sample size contributes to develop an equation of fat free mass specific to dialysis patients.

## Conclusion

In conclusion, this study provides FFM results of healthy adults and dialysis patients by different BIA equations, suggesting that equations developed by healthy subjects might be not appropriate for dialysis patients, especially peritoneal dialysis patients. It is recommended to develop a specific BIA equation from dialysis population.
